# Structural and functional basis for RNA cleavage by Ire1

**DOI:** 10.1186/1741-7007-9-47

**Published:** 2011-07-06

**Authors:** Alexei V Korennykh, Andrei A Korostelev, Pascal F Egea, Janet Finer-Moore, Robert M Stroud, Chao Zhang, Kevan M Shokat, Peter Walter

**Affiliations:** 1 Howard Hughes Medical Institute, University Of California, San Francisco, Genentech Hall, 600-16th Street, San Francisco, CA 94158, USA; 2 Department of Biochemistry and Biophysics, University of California, San Francisco, Genentech Hall, 600-16th Street, San Francisco, CA 94158, USA; 3 Department of Biochemistry and Molecular Pharmacology, and RNA Therapeutics Institute, University of Massachusetts Medical School, Worcester, MA 01605, USA; 4 Department of Cellular and Molecular Pharmacology, University of California, San Francisco, Genentech Hall, 600-16th Street, San Francisco, CA 94158, USA; 5 Department of Molecular Biology, Princeton University, 216 Schultz Laboratory, Princeton, NJ 08544, USA; 6 Department of Biological Chemistry, University of California, Los Angeles, 310 BSRB, Los Angeles, CA 90095, USA

## Abstract

**Background:**

The unfolded protein response (UPR) controls the protein folding capacity of the endoplasmic reticulum (ER). Central to this signaling pathway is the ER-resident bifunctional transmembrane kinase/endoribonuclease Ire1. The endoribonuclease (RNase) domain of Ire1 initiates a non-conventional mRNA splicing reaction, leading to the production of a transcription factor that controls UPR target genes. The mRNA splicing reaction is an obligatory step of Ire1 signaling, yet its mechanism has remained poorly understood due to the absence of substrate-bound crystal structures of Ire1, the lack of structural similarity between Ire1 and other RNases, and a scarcity of quantitative enzymological data. Here, we experimentally define the active site of Ire1 RNase and quantitatively evaluate the contribution of the key active site residues to catalysis.

**Results:**

This analysis and two new crystal structures suggest that Ire1 RNase uses histidine H1061 and tyrosine Y1043 as the general acid-general base pair contributing ≥ 7.6 kcal/mol and 1.4 kcal/mol to transition state stabilization, respectively, and asparagine N1057 and arginine R1056 for coordination of the scissile phosphate. Investigation of the stem-loop recognition revealed that additionally to the stem-loops derived from the classic Ire1 substrates *HAC1 *and *Xbp1 *mRNA, Ire1 can site-specifically and rapidly cleave anticodon stem-loop (ASL) of unmodified tRNA^Phe^, extending known substrate specificity of Ire1 RNase.

**Conclusions:**

Our data define the catalytic center of Ire1 RNase and suggest a mechanism of RNA cleavage: each RNase monomer apparently contains a separate catalytic apparatus for RNA cleavage, whereas two RNase subunits contribute to RNA stem-loop docking. Conservation of the key residues among Ire1 homologues suggests that the mechanism elucidated here for yeast Ire1 applies to Ire1 in metazoan cells, and to the only known Ire1 homologue RNase L.

## Background

The unfolded protein response (UPR) is an intracellular signaling pathway that provides homeostatic feedback regulation between the endoplasmic reticulum (ER) and the gene expression program in the nucleus. To this end, the UPR senses the conditions inside the ER, detecting an imbalance between newly made proteins and the protein folding capacity in the ER, and activates a corrective response. For signaling, the UPR uses a transmembrane sensor of ER-lumenal unfolded proteins, Ire1.

Ire1 is an ER membrane-resident receptor that serves as a primary signal transduction device in the UPR conserved from yeast to mammalian cells [1-4]. Oligomerization of Ire1-lumenal domains is thought to be a key event in initiating signal propagation across the ER membrane that enables the cooperative assembly of Ire1's cytosolic kinase and RNase modules into an ordered oligomer with a defined three-dimensional structure [5]. The oligomer is stabilized by phosphates resulting from autophosphorylation of the kinase domain and allows juxtaposition of Ire1's RNase domains, which presumably activate the RNase [5,6]. Once activated, Ire1 initiates the non-conventional splicing of *HAC1 *mRNA (yeast) or *XBP1 *mRNA (metazoan) by cleaving the mRNA at two conserved sites to excise an intron [5,7]. An RNA ligase (tRNA ligase in yeast and a still unknown enzyme in metazoan cells) rejoins the severed exons to complete the reaction. Intron removal allows for the production of the UPR transcription activators Hac1 and XBP1, respectively, which upregulate UPR target genes.

Ire1's RNase domain thereby serves the primary role in signal transmission. The mechanism underlying activation of Ire1 RNase by oligomerization and the mechanism of mRNA recognition and cleavage have remained elusive. To date, there are no known structural homologues of the Ire1 RNase domain that could help answering these questions. The best attempt at defining the mechanism of RNA cleavage has been made based on sequence conservation arguments and on a crystal structure of Ire1 dimer with a ligand-free RNase domain, which also lacked a fragment of the RNase active site [6]. In this work, the RNase dimer was proposed to contain two independent catalytic centers, one per Ire1 monomer, which would simultaneously accommodate the two RNA stem-loops conserved in all known mRNA substrates of Ire1 [6]. According to the proposed model, two stem-loops would form a kissing interaction for docking and recognition by Ire1. Subsequent studies proposed [5] that such a kissing interaction is not likely because Ire1 exhibits no preference for RNA substrates with dual stem-loops over substrates with a single stem-loop, indicating that a single stem-loop structure is the cognate folded substrate of Ire1 RNase.

It has been also suggested [6] that putative active site residues are positioned similarly in Ire1 RNase and in pre-tRNA splicing endonuclease (SEN), although these two endoribonucleases share no apparent sequence or structural homology and cleave dissimilar RNA substrates (stem-loop versus bulge-helix-bulge motif). The authors point out [6] that the putative catalytic residues in Ire1 do not align completely with those in SEN and would require a 5-Å displacement of Y1043 (in yeast Ire1) to bring it into a position corresponding to that of Y249 in SEN. A 5-Å conformational change in Ire1 RNase upon substrate binding has been proposed based on these arguments [6]. A new cocrystal structure of Ire1 RNase with an oligonucleotide bound, which we provide in this work, does not support considerable conformational changes in the position of Y1043. Therefore, Ire1 and SEN apparently cleave RNA using different arrangements of active site residues. Underscoring this difference, substitution of a single catalytic histidine residue H1061 produced orders-of-magnitude greater effect on the catalytic activity of Ire1 (this work) compared to only a 28-fold rate reduction in SEN [8].

Conservation of residue H1061, the only invariant histidine in the RNase domain of Ire1, was used to propose that H1061 marks the catalytic center and serves for general acid-general base catalysis [6]. In agreement with this model, mutation H1061A reduced RNA cleavage [6]. However, this mutation was analyzed using a qualitative approach that had a narrow dynamic range and did not distinguish between effects on binding and catalysis. Furthermore, mutation H1061A not only removed the ability of histidine to transfer protons but also the ability to form hydrogen bonds, which alone could explain the observed effect on RNA cleavage, without invoking H1061 in general acid-general base catalysis. Considering that ribonucleases can cleave RNA stem-loops by using for proton transfer basic amino acids instead of conventional ionizable residues (histidine, glutamate and aspartate) [9], the very involvement of H1061 in general acid-general base catalysis could not be deduced convincingly from any of the available data.

To understand the mechanism of RNA cleavage by Ire1, we combined quantitative analysis of rationally designed Ire1 mutants and X-ray crystallography. We show that proton transfer by histidine H1061 contributes greater than five orders of magnitude (≥ 7.6 kcal/mol) to catalysis of RNA cleavage, which experimentally defines a key catalytic functionality in the active center of Ire1 RNase. Using the non-disruptive Y1043F mutation we experimentally demonstrate a catalytic role of the OH group of Y1043 and also show an important role in RNA binding of a helix-loop element (HLE) residue, R1039. Our findings suggest parallels between catalytic centers of Ire1 and the well characterized RNases A and T1 and propose an unexpected mechanism of oligomerization-induced *in trans *RNA recognition by Ire1.

## Results

### Computational analysis of the RNA binding properties of Ire1

**Figure 1:**
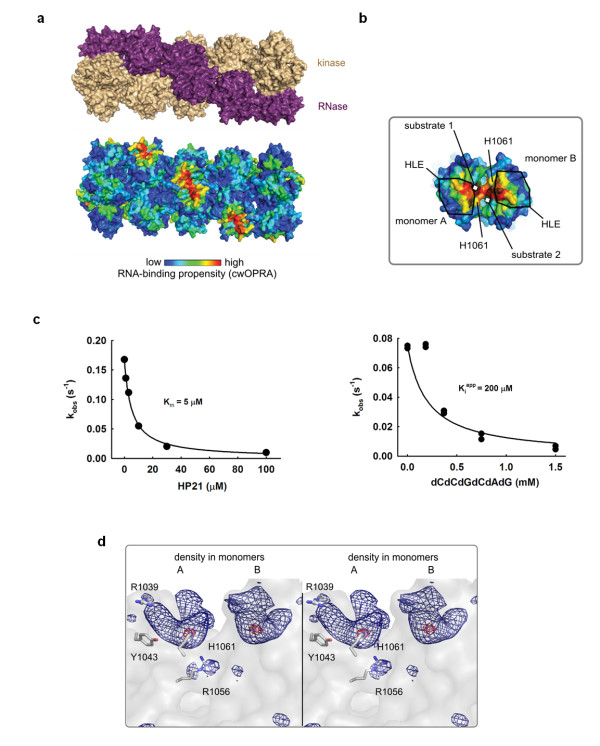
**Oligonucleotide binding to Ire1 RNase**. **(a) **Ire1 oligomer (PDB ID 3fbv) colored by domains (top) and RNA-binding propensity (bottom). **(b) **Close view of symmetric back-to-back RNase dimer from the oligomer in (a) with electron density for dCdCdGdCdAdG from the C222 crystal structure superimposed. Electron density colocalizes with the area of the strongest RNA binding propensity and is in direct contact with conserved histidine H1061 and the helix-loop element (HLE). **(c) **Binding of an RNA stem-loop HP21 and of dCdCdGdCdAdG to Ire1KR32 measured via inhibition of ^32^P-5'-HP21 cleavage by unlabeled HP21 (K_m _= 5 ± 1 μM) or dCdCdGdCdAdG (K_i_^app ^= 200 ± 50 μM). Reactions contained 20 mM 4-(2-hydroxyethyl)-1-piperazine-ethanesulfonic acid (HEPES) (pH 7.4), 70 mM NaCl, 5% glycerol, 2 mM Mg(CH_3_COO)_2_, 2 mM ADP, 4 mM dithiothreitol, 3 μM total Ire1KR32 and ≤ 1 pM ^32^P-5'-HP21, and were conducted at 30°C. Measurements of HP21 binding at 1 μM and 10 μM of total Ire1KR32 are shown in Additional file 1, Figure S1. **(d)**, Wall-eyed stereoview of non-crystallographic symmetry (NCS)-averaged F_o_-F_c _electron density map at the RNase active site. Contour levels are 7σ (dark blue) and 13σ (red). Side chain positions are from the crystal structure 3fbv.

To glean insights into Ire1 interactions with *HAC1*/*XBP1 *mRNA, we analyzed the RNA binding properties of the entire surface of the Ire1 oligomer [5]. Surface electrostatics calculations [10] are commonly used for such analyses, but they produce ambiguous results because electrostatics is not the sole determinant of RNA binding. Here we used a modified optimal protein-RNA area (OPRA) algorithm that previously demonstrated high prediction accuracy [11]. We refined this method by adding phylogenetic conservation to the scores and termed the resulting algorithm 'conservation-weighted OPRA' (cwOPRA; to be described elsewhere). The cwOPRA analysis of RNA binding properties of the Ire1 oligomer produced a high-contrast image of the surface RNA binding propensity. A single RNA binding site was strongly predicted in the center of every back-to-back RNase dimer [6]. The RNA binding site is proximal to the dynamic helix-loop element HLE, which becomes more ordered upon formation of Ire1/Ire1 interfaces in the high-order oligomer and has been proposed to serve as a part of the active site [5] (Figure 1a, b). The rest of the Ire1 surface was predicted not to interact with RNA (we note, however, that the crystal structure is lacking a highly basic N-terminus, which is disordered and could serve as a secondary RNA binding site). We next validated this computational prediction using a cocrystal structure of Ire1 with an oligonucleotide.

### Crystal structure of Ire1 with dCdCdGdCdAdG bound to the RNase domain

For crystallization trials, we tested a panel of RNase-resistant RNA analogs with 2'-deoxy or 2'-methoxy substitutions throughout and a variety of Ire1 constructs. Only a single combination led to formation of diffracting crystals as discussed below. Attempts to soak mononucleotides, dinucleotides, trinucleotides or oligonucleotides (0.5-20 mM) into previously characterized Ire1 crystals [5] were unsuccessful. No additional difference density was observed, which could correspond to the soaked-in substrate analogs.

By contrast, cocrystallization of Ire1KR32 with a 6-mer 2'-deoxy-substituted oligonucleotide bearing the consensus sequence of an Ire1 splice site CCGCAG (the use of 2'-deoxy substitutions prevents degradation by Ire1 RNase) resulted in crystals of the Ire1KR32·dCdCdGdCdAdG complex. Ire1KR32 construct contains the Ire1's kinase domain, the RNase domain, and a 32-amino-acid linker previously shown to be required for optimal RNase activity [5]. The dCdCdGdCdAdG oligonucleotide bound to Ire1KR32 with K_i_^app ^= 200 μM determined from inhibition of the Ire1KR32-catalyzed cleavage of HP21, a small stem/loop structure containing the 3' splice site of *XBP1 *mRNA [5]. This value is approximately 40-fold weaker than that for the cognate stem-loop (K_m _= 5 μM, Figure 1c and Additional file 1, Figure S1) possibly due to a more flexible structure of the single-stranded oligonucleotide dCdCdGdCdAdG compared to HP21 RNA stem-loop and/or due to the lack of 2'OH groups. Unlike the oligonucleotide-free Ire1, which crystallizes in space group P2_1_2_1_2 [5], the Ire1KR32·dCdCdGdCdAdG oligomer crystallized in space group C222 and diffracted to 6.6 Å (Additional file 1, Table S1). The presence of the oligonucleotide allowed Ire1KR32 to crystallize without a ligand occupying the kinase ATP binding pocket, providing an important insight into the mechanism of cofactor-dependent activation of Ire1 [12].

We used the 3.2-Å structure of Ire1 crystallized in the absence of a nucleic acid substrate [5] for molecular replacement and subsequent rigid-body refinement, as described in Methods and in the accompanying manuscript [12]. At 6-7 Å resolution, the secondary structure of proteins and the sugar-phosphate backbone, which is the most electron-dense part of nucleic acids, can be visualized in ribonucleoprotein complexes [13,14]. Accordingly, we observed a strong F_o_-F_c _difference electron density at the core of the RNase domain, which coincided with the cwOPRA-predicted RNA binding site (Figure 1b). The electron density was delineated by two separate contours related by twofold symmetry (Figure 1b, d). Each contour (at level 7σ) defines an elongated volume of approximately 20 Å in length, consistent with the length of one dCdCdGdCdAdG molecule bound per RNase monomer. We placed Ire1 side chains according to the 3.2-Å-resolution search model used for the molecular replacement (Figure 1d). Such an approximation is valid because the protein backbone traces and the quaternary structure of Ire1 oligomers superimpose in the 6.6Å and the 3.2Å structures, even though they crystallized in different space groups (P2_1_2_1_2 vs C222). Based on the complete superposition of the polypeptide backbones in Ire1KR32·APY29 [5] and in Ire1·dCdCdGdCdAdG complexes, we can rule out the possibility of a large-scale movement in the catalytic core of Ire1 upon binding of the substrate analog.

### Probing the catalytic mechanism of Ire1 RNase via mutagenesis: role of H1061

The electron density for dCdCdGdCdAdG is located in the cleft formed by the HLEs [5]. The position of the electron density suggests that eight protonatable residues of Ire1 located proximal to the substrate mimic could, in principle, contribute to general acid-general base catalysis leading to the formation of the 2', 3'-cyclic phosphate [15]. Because Ire1 does not require divalent metal ions for RNase activity [5], it must rely exclusively on amino acid side chains to carry out catalysis. In the identified group of amino acids, only H1061 and D1064 have pK_a _values near neutrality, analogous to the catalytic residues involved in proton transfer in well characterized ribonucleases, such as RNase A [16] and RNase T1 [17]. The H1061 is invariant in all known Ire1 sequences and the only Ire1 homologue RNase L [18]. By contrast, D1064 is mutated in several organisms to residues incapable of proton transfer (Additional file 1, Table S2), indicating that it is dispensable for catalysis.

**Figure 2:**
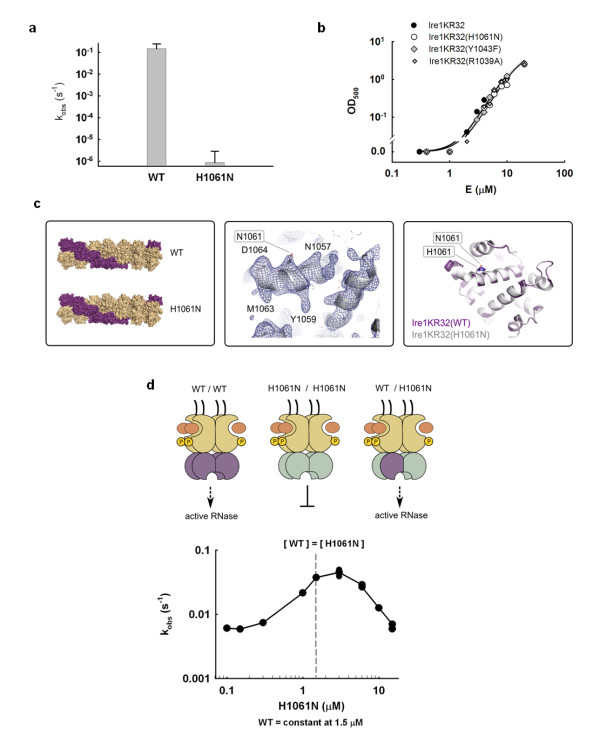
**Probing the role of H1061 in catalysis**. **(a) **Effect of H1061N mutation on the RNase activity of Ire1. Error bars show standard error of single-exponential fitting. **(b) **Oligomerization profiles of Ire1KR32 and three mutants, Ire1KR32(H1061N), Ire1KR32(Y1043F) and Ire1KR32(R1039A) measured using light absorbance at 500 nM (see Methods). Measurements were conducted at room temperature using the same reaction buffers as in Figure 1c. **(c) **Comparison of Ire1 structures from oligomers formed by wild-type Ire1 (PDB ID 3fbv, space group P2_1_2_1_2) and by H1061N mutant (new 3.65Å crystal structure, also in space group P2_1_2_1_2). Shown are overall oligomer architectures (left), 2F_o_-F_c _electron density map for the H1061N RNase domain contoured at 1.3σ (middle) and superposition of the RNase domains (right). Simulated annealing F_o_-F_c _omit map for H1061N calculated without non-crystallographic symmetry (NCS) is shown in Additional file 1, Figure S2. **(d) ***In trans *activation of Ire1KR32 by the non-catalytic mutant H1061N. Concentration of wild-type Ire1KR32 was 1.5 μM, concentration of the H1061N Ire1 varied between 0.1-15 μM. Arrow marks the point of equivalent concentrations. Reactions were conducted under single-turnover conditions using the same reaction buffer as in Figure 1c.

Mutation of the catalytic histidine to alanine, asparagine or glutamine diminishes catalysis by ribonucleases T1 and A by 10^2 ^to 10^4^-fold [17,19,20]. In a previous study, an H1061A mutation in Ire1 was reported to inhibit the RNase activity; however, the quantitative effect of this mutation on catalysis has not been measured [6]. To determine the quantitative contribution of H1061 to catalysis, we expressed and purified a mutant of Ire1KR32 in which H1061 was replaced by asparagine. We chose mutation to asparagine because it best preserves the space filling and hydrogen bonding properties of histidine, while removing histidine's ability to transfer protons and to support catalysis. We observed that Ire1KR32(H1061N) displays a ≥ 3 × 10^5^-fold rate reduction compared to wild-type Ire1KR32 (Figure 2a), strongly indicating that H1061 plays a central part in catalysis.

Next, we ruled out a possibility that the large effect of the H1061N mutation arose from a perturbation of oligomerization. To this end, we monitored the optical density of Ire1KR32(H1061N) solutions at 500 nm, which provides a quantitative measure of Ire1KR32 oligomerization (Methods and [12]). Ire1KR32(H1061N) mutant exhibited an indistinguishable oligomerization profile from that of wild-type Ire1KR32 (Figure 2b), suggesting that the H1061N mutation does not interfere with oligomer formation.

To further ascertain that the dramatic rate reduction does not arise from more local conformational perturbations of Ire1 RNase, uncoupled from oligomerization, we crystallized Ire1KR32(H1061N) in complex with the synthetic ligand APY29, which binds in Ire1's kinase ATP binding pocket [5]. We determined a 3.65-Å-resolution crystal structure of this mutant (Additional file 1, Table S3), which proved identical to that of wild-type Ire1KR32 (Figure 2c; Additional file 1, Figure S2). Our functional and structural results pertaining to H1061N mutant therefore indicate that the greater than 3 × 10^5^-fold catalytic impairment of Ire1KR32(H1061N) arises from a direct role of H1061 in proton transfer during RNA cleavage.

Due to the twofold symmetry of the Ire1 RNase dimer [6], a single H1061N mutation removes two H1061 residues in the vicinity of the RNase active site (Figure 1b). We thus wondered whether the catalytic impairment of the H1061N mutant might arise from this symmetry: if Ire1 required both H1061 residues together to catalyze phosphodiester cleavage, we would *de facto *have measured activity of a double mutant. To explore this possibility, we built Ire1 oligomers in which each catalytic H1061 would be paired with a non-catalytic H1061N in chimeric RNase dimers. To this end, we titrated catalytically inactive Ire1KR32(H1061N) into a solution of wild-type Ire1KR32 held at a constant concentration just below its oligomerization threshold. For enzymes that follow Michaelis-Menten kinetics, addition of a catalytically inactive mutant to a wild-type reaction inhibits the rate, as inactive enzyme sequesters the available substrate. By contrast, we observed approximately tenfold activation of Ire1KR32 before onset of inhibition at excessive concentrations of Ire1KR32(H1061N) (Figure 2d). Stimulation occurs because wild-type Ire1KR32 becomes trapped in a wild-type/H1061N hetero-oligomer as enzyme concentration increases and causes Ire1 to oligomerize. Kinetic modeling of this experiment shows that such a response is expected only if wild-type:H1061N heterodimers in the Ire1KR32 oligomer were active RNases (Additional file 1, Figure S3 and Additional file 2). We conclude that only one of the two H1061 residues in the twofold symmetric RNase dimer is required for RNA cleavage.

**Figure 3:**
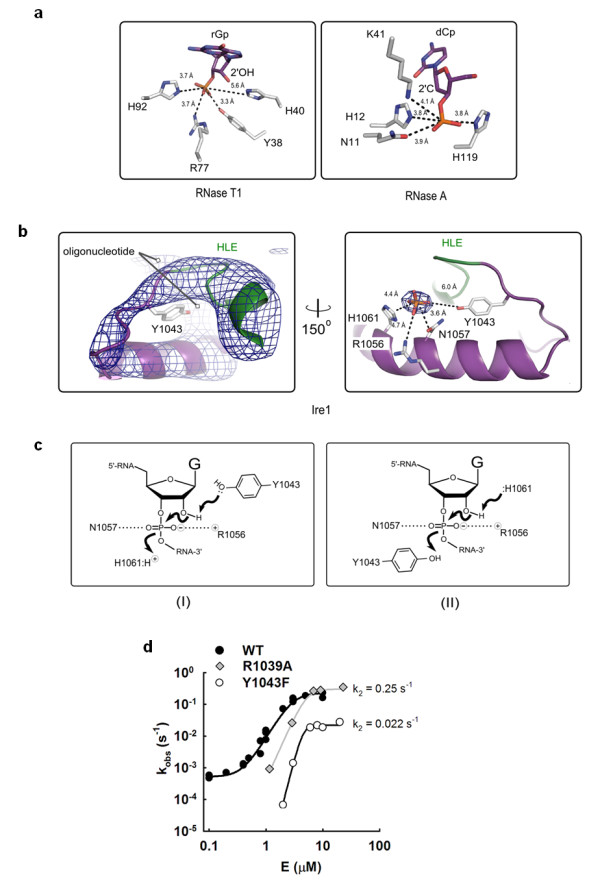
**Catalytic mechanism of Ire1 RNase**. **(a) **Catalytic residues of RNase T1 and RNase A with productively bound nucleotides (PDB ID 1r5c and 1rga). **(b) **Left panel: simulated annealing (1000 K) omit-electron density map F_o_-F_c _for the helix-loop element (HLE; green) and adjacent loop containing Y1043 and the helix α4 of RNase domain (contoured at 5σ). Refinement was conducted with residues 1032-1057 deleted from all 7 monomers in the asymmetric unit, without using non-crystallographic symmetry (NCS). An alternative view of the electron density (after rotation around vertical axis) is shown in Additional file 1, Figure S4. Right panel: position of the scissile phosphate in the active site of Ire1 RNase. The strongest peak of the electron density (F_o_-F_c_, contoured at 12σ) was used to position the phosphate. Side chains were taken from PDB ID 3fbv). **(c) **Two plausible catalytic mechanisms in the active site of Ire1 RNase. **(d) **Enzyme titration profiles for Ire1KR32, Ire1KR32(Y1043F) and for Ire1KR32(R1039A) under single-turnover conditions. Reactions were conducted as in Figure 1c.

Proximity to the electron density of the substrate, mutagenesis analysis, evolutionary conservation and the neutral pK_a _value of histidine all converge on the suggestion that H1061 serves a catalytic function. Cleavage of an RNA backbone by other non-metal RNases involves pairs of conserved residues that carry out general acid-general base catalysis and lie at a fixed mutual separation of 6-8 Å (for example, PDB ID 1r5c, 1rga; Figure 3a). In Ire1, only two invariant amino acids, R1056 and Y1043, are located 5.5 Å and 8 Å from H1061, respectively (Additional file 1, Table S2), and hence emerge as candidates to be a catalytic partner of H1061. Steric considerations discussed below and high pK_a _value of arginine argue that R1056 is unlikely to fulfill this role, suggesting Y1043 as the most plausible partner of H1061 (Figure 3b, c; see Discussion). We note that the R1056 side chain is deeply buried in the active site, which would make it difficult to interpret results of mutational analyses. We therefore did not pursue analysis of R1056 at this time.

### Probing the role of Y1043 as the catalytic partner of H1061

Previously, it was suggested that if Ire1 RNase resembled SEN endoribonuclease, then tyrosine Y1043 may serve as the general base [6]. The fact that Y1043 resides approximately 5 Å from the analogous tyrosine in SEN was used to propose that Ire1 RNase should undergo an obligate conformational change upon binding of substrate to position Y1043 relative to H1061 as in SEN. To test this possibility, we examined the electron density for the Y1043-bearing loop in the Ire1KR32·dCdCdGdCdAdG complex (Figure 3b; Additional file 1, Figure S4). We found that backbones of the HLE and of the adjacent Y1043-bearing loop occupy the same position as in the 3.2-Å crystal structure obtained without bound oligonucleotide (PDB ID 3fbv). These findings support a model that tyrosine Y1043 does not move over a significant distance upon substrate binding.

To assess whether Y1043 contributes to catalysis, we prepared and quantitatively characterized the mutant Ire1KR32(Y1043F), which is predicted to disable any involvement of Y1043 in proton transfer while ensuring minimal structural perturbation to the protein compared to a potentially far more disruptive Y1043A mutation tested previously [6]. We obtained an activation profile with Ire1KR32(Y1043F) (Figure 3d, open circles). At enzyme concentrations exceeding 3 μM, the observed rate constant for cleavage of HP21 RNA plateaued at a first-order single-turnover rate constant k_2 _= 0.022 s^-1^. This value is approximately tenfold smaller than the corresponding value for wild-type Ire1KR32 (k_2 _= 0.25 s^-1^), demonstrating that the OH group of Y1043 is important for catalysis. Similar to the H1061N mutation, the Y1043F mutation had no effect on the oligomerization equilibrium of Ire1KR32 (Figure 2b). The deleterious effect of the Y1043F mutation was greater at subsaturating k_2_/K_1/2 _conditions encountered below approximately 3 μM enzyme concentrations, indicating that Y1043 also contributes to binding of the RNA substrate (Figure 3d).

### Probing the role of the HLE in RNA recognition

Location of the dCdCdGdCdAdG electron density in proximity to the HLE (Figure 1b, d) confirms the previously posed hypothesis [5,6] that this element serves as an important part of the active site. It was proposed that residues from HLEs could serve for binding and recognition of RNA substrates, rather than for catalysis. Mutations in the HLE are therefore expected to provide a valuable control to contrast the catalytically important mutations of H1061 and Y1043 described above. To test the role of the HLE in RNA cleavage, we prepared Ire1KR32 with a mutation of an arginine residue within the HLE, Ire1KR32(R1039A) and measured the titration rate profile for the Ire1KR32(R1039A) mutant (Figure 3d, diamonds). At saturating enzyme concentrations Ire1KR32(R1039A) achieved the same first-order single-turnover rate constant k_2 _as did wild-type Ire1KR32 (Figure 3d, closed circles), indicating that R1039 makes no contribution to stabilization of the transition state. Under subsaturating conditions, however, the R1039A mutant became deleterious, revealing a role of R1039 in substrate binding. Analogous to other mutations tested here, Ire1KR32(R1039A) exhibited an unperturbed oligomerization profile (Figure 2b), indicating that this mutation too does not affect the enzyme's oligomerization equilibrium. Both structural (Figure 1d) and functional data with Ire1KR32(R1039A) therefore show that the HLE plays an important role in recognition of RNA substrates.

**Figure 4:**
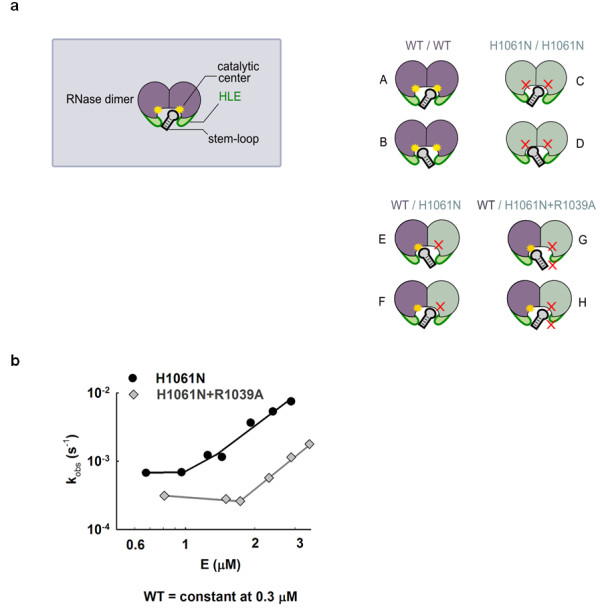
***In trans *activation of wild-type Ire1 RNase by Ire1 with RNase with a double mutation**. **(a) **Schematic representation of RNase dimers formed via interface IF1^c ^[5]. Due to a twofold symmetry, each RNase dimer can accommodate RNA stem-loop in two equivalent orientations (such as A and B). Both orientations are productive for wild-type Ire1. Neither orientation is productive for the H1061N mutant (C) and (D) due to disrupted catalysis. Only one orientation (E) is productive for the wild-type/H1061N chimera. Only one orientation (G) is also productive for the wild-type/H1061N+R1039A chimera, however this orientation is impaired by the R1039A mutation in the helix-loop element (HLE). **(b) **Titration of wild-type Ire1KR32 with Ire1KR32(H1061N) single mutant and Ire1KR32(H1061N, R1039A) double mutant. Reactions were conducted as in Figure 2d but contained 0.3 μM wild-type Ire1KR32 throughout the titrations to ensure subsaturating (k_2_/K_1/2_) regime for RNA cleavage.

Electron density-guided docking of an RNA stem-loop into Ire1 RNase (below) suggests that the HLE of one RNase monomer may contribute to RNA cleavage in the active site of an adjacent RNase monomer, acting *in trans*. To test this possibility, we performed *in trans *activation experiments as in Figure 2d, in which we analyzed the activity of Ire1KR32(wild-type)/Ire1KR32(R1039A) heterodimers. To ensure that effect of the R1039A mutation is indeed measured *in trans*, we additionally disabled the catalytic site of the R1039A mutant by the H1061N mutation, as outlined in Figure 4a. The experiment was conducted as in Figure 2d, but contained lower concentration of wild-type Ire1KR32 (0.3 μM or approximately 0.06 K_m_) to ascertain a regime in which subsaturating conditions (k_2_/K_1/2_) were maintained during the titration. This was an important consideration because if the reactions were allowed to approach the first-order k_2 _regime, the deleterious effect of the R1039A mutation on binding would have been masked (note loss of effect from the R1039A mutation in Figure 3d when the reaction plateaued at E_0 _> 5 μM). Using this experiment, we found that the RNase activity of wild-type Ire1KR32 stimulated *in trans *by the double mutant Ire1KR32(R1039A, H1061N) was an order-of-magnitude lower than with the H1061N single mutant (Figure 4b). The chimeric dimer with the double mutation (G in Figure 4a) therefore binds RNA more weakly than the chimeric dimer with the single mutation (E in Figure 4a), whereas the only difference between these two cases is an intact HLE (E) versus a HLE bearing a single R1039A mutation (G). It follows that the RNA cleavage reaction catalyzed by one RNase monomer is sensitive to a mutation in the HLE of the paired monomer. These observations strongly support a model of a composite RNA-binding surface in Ire1 RNase, which also emerges from the electron density-guided RNA stem-loop docking described below.

### Ire1 site-specifically cleaves stem-loop anticodon of tRNA^Phe^

**Figure 5:**
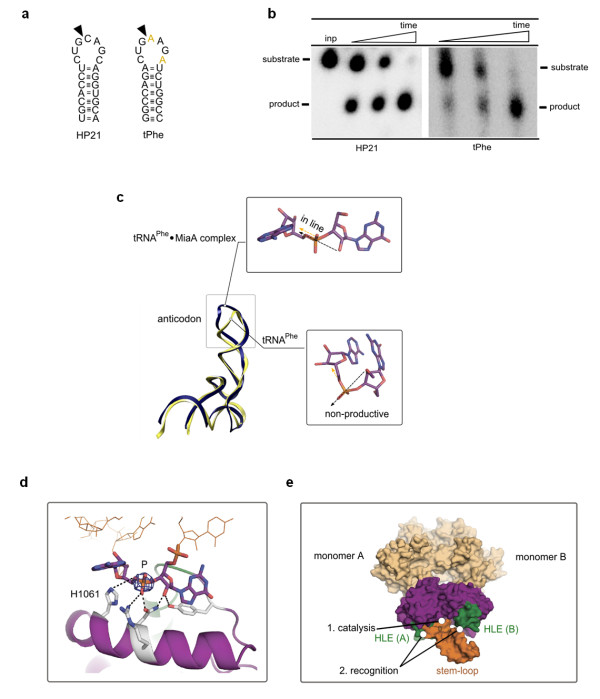
**Model of stem-loop recognition via a composite protein surface**. **(a) **Stem-loops derived from XBP1 mRNA (HP21) and phenylalanine tRNA^Phe ^(tPhe). Loop sequence differences are colored yellow in tPhe. **(b) **Time courses (0-5 min) for cleavage of 5'-^32^P-labeled stem-loops HP21 and tPhe by Ire1KR32 (3 μM) analyzed by polyacrylamide gel electrophoresis. **(c) **Superposition of crystal structures of tRNA^Phe ^(PDB ID 1ehz) and tRNA^Phe ^from tRNA^Phe^·MiaA complex (PDB ID 2zm5). Black arrow shows direction of nucleophile attack, orange arrow shows direction of leaving group. **(d) **Manual rigid-body docking of the base-flipped tRNA^Phe ^anticodon into Ire1 crystal structure. Electron density for the scissile phosphate (contoured at 12σ) and the proposed catalytic mechanism (Figure 3c-I) were used as docking constraints. P designates scissile phosphate. The helix-loop element (HLE) is colored green. **(e) **Surface rendering of the model in (d). The model suggests cleavage of a stem-loop by one active site and recognition by a composite RNA binding surface.

Ire1 recognizes and site-specifically cleaves conserved sites in *HAC1 *and *XBP1 *mRNA, which are characterized by a stem containing approximately five Watson-Crick base pairs and a seven-residue loop with a consensus sequence CNGNNGN [21]. To understand how Ire1 might recognize such stem-loops, we searched bioinformatically for RNA heptaloops with similar features. This search showed that the anticodon stem-loop of tRNA^Phe ^closely resembles the Ire1 consensus substrate (Figure 5a). To verify that this sequence similarity meaningfully identifies an Ire1 substrate, we conducted RNA cleavage assays and compared the kinetics of cleavage by Ire1 of tRNA^Phe ^anticodon stem-loop and cleavage of the stem-loop derived from *XBP1 *mRNA, HP21. Ire1 cleaved both stem-loops in a single site and with similar rates (Figure 5b), indicating that the tRNA^Phe ^anticodon stem-loop indeed provides a good model of the cognate Ire1 stem-loops from *XBP1 *and *HAC1 *mRNA. As multiple crystal structures of tRNA^Phe ^have been already solved, this finding enabled us to model the docking of an RNA stem-loop to the Ire1 RNase active site (Figure 5c-e).

## Discussion

### The architecture of Ire1 RNase

Our conclusion that each Ire1 monomer contains a complete catalytic center in the context of the Ire1 oligomer agrees with the model proposed previously based on experiments in which several Ire1 variants with dissimilar RNase-inactivating mutations were mixed [6]. The previous observation showed that none of the mutant mixtures have RNase activity and was interpreted in favor of individual active sites over a composite catalytic center (created by association of two RNase monomers) [6]. Importantly, however, this lack of RNase activity can be reasonably reconciled with both, per monomer and composite active site architectures, as well as with a third possibility of indirect defects in Ire1 RNase due to misfolding or conformational perturbation of the mutants. By contrast, the observation of electron density for a substrate mimic in each RNase monomer (Figure 1b, d) and *in trans *activation of wild-type Ire1KR32 by Ire1KR32(H1061N) (Figure 2d) provide strong experimental support for an individual catalytic center in each RNase monomer.

### Position of the scissile phosphate and the catalytic mechanism of Ire1 RNase

We deduce the position of the scissile phosphate of a *bona fide *Ire1 RNA substrate in this catalytic center using the structure of the Ire1KR32 complexed with a DNA hexanucleotide, dCdCdGdCdAdG, which mimics the cognate Ire1 substrate. This substrate analog was chosen due to present experimental limitations imposed by the inability of Ire1 to cocrystallize with other oligonucleotides. While we recognize that the deoxy-substituted single-stranded substrate may be structurally distinct from RNA stem-loops, we surmise that its binding site on Ire1 represents that of the HP21 RNA because (i) dCdCdGdCdAdG efficiently inhibits cleavage of HP21 by Ire1KR32, and (ii) the electron density in the RNase active site directly contacts the critical catalytic residue H1061, which is involved in proton transfer and contributes ≥ 3 × 10^5^-fold to catalysis. At high contour levels, we could identify three near-spherical peaks in a F_o_-F_c _Fourier map. The central peak was strongest and was visible in most Ire1 monomers in the asymmetric unit, while the other two peaks could be observed in several but not all Ire1 monomers. Because the central peak most likely represents the most-ordered phosphate group of the oligonucleotide and is located between the functionally validated catalytic residues H1061 and Y1043 (Figures 2a and 3d), we suggest that it corresponds to the position of the scissile phosphate of the substrate.

Placement of this phosphate and comparison of the active site of Ire1 with the active sites of RNase T1 and A suggest putative roles of Ire1 residues in catalysis (Figure 3a, b). Similar to Ire1, ribonucleases T1 and A do not use metal ions [5] and produce 2',3'-cyclic phosphate [21]. Importantly, the mechanisms of RNA cleavage have been established in detail for both RNase T1 and RNase A [20,22-24].

Structural comparison shows that Ire1 and RNases T1 and A have a single positively charged residue contacting the phosphate: R1056 (Ire1), R77 (RNase T1) and K41 (RNase A). These positively charged residues serve for catalysis by stabilizing the negative charge buildup on non-bridging oxygens in the transition state, as has been shown for RNase A [20]. By analogy, R1056 of Ire1 may also participate in transition state stabilization and is unlikely to directly participate in proton transfer. The last notion is supported by steric considerations: R1056-phosphate-H1061 connectivity would form an atypically acute angle for a general acid-phosphate-general base triad, placing phosphate entirely off the line connecting the two protein residues implicated in proton transfer (compare Figure 3a and 3b). This arrangement would not be compatible with in-line geometry required for RNA cleavage to form a 2',3'-cyclic product.

In RNase T1 and RNase A, a single uncharged hydrophilic residue is positioned off the line connecting general acid, scissile phosphate and general base, and at a hydrogen-bonding distance from the phosphate oxygens: Y38 in RNase T1 and N11 in RNase A, respectively. In RNases T1 and A, these residues play a catalytic role by stabilizing the transition state via coordination of non-bridging oxygen [25]. Position of the electron density in Ire1 (Figure 3b, right panel) predicts an analogous catalytic function for N1057.

Finally, all three RNases contain two residues that act as a general acid/general base pair. For RNases T1 and A, general acids are H92 and H119 at a distance of 3.7 and 3.8 Å from the scissile phosphate, respectively. General bases are H40 and H12 at a distance 5.6 Å and 3.8 Å, respectively. The position of the electron density for the phosphate between the H1061 and Y1043 (Figure 3b) agrees with the role of these residues in Ire1 as the general acid/general base pair (Figure 3c). Whereas the role of H1061 parallels that of the histidines in RNase T1 and A, a catalytic tyrosine is unusual because of the high pK_a _of approximately 10. Experimental testing of the catalytic role of Y1043 using an Y1043F mutation revealed that the hydroxy group of Y1043 contributes a rate enhancement of approximately tenfold (or approximately 1.4 kcal/mol) to catalysis. Our data therefore are in agreement with the catalytic role of Y1043 and suggest two kinetically equivalent catalytic mechanisms shown in Figure 3c. We note that the tenfold transition-state effect of the Y1043F mutation is modest and thus it could also be consistent with a third possibility that the OH group of Y1043 plays an indirect role in catalysis and forms an important hydrogen-bonding network in the active site rather than participates in proton transfer directly. This alternative possibility cannot be ruled out until an atomic resolution cocrystal structure of an Ire1-RNA complex becomes available. If Y1043 serves for proton transfer directly, then its relatively small catalytic contribution compared to the ≥ 3 × 10^5^-fold contribution of the partnering H1061 residue or the 10^3 ^to 10^4^-fold contributions of the catalytic histidines in RNase A or T1 [17,20] could be accounted for quantitatively by the 10^3 ^to 10^4 ^higher pK_a _of tyrosine compared to histidine.

While our data support the roles of the H1061 and Y1043 as a general acid/general base catalytic pair, they do not unambiguously define which residue serves as the general acid or the general base. Such an assignment has proven challenging for RNases and ribozymes, even when high-resolution cocrystal structures with substrate analogs or products were available [20,26]. At present, we cannot formally distinguish between the two plausible mechanisms for RNA cleavage by Ire1 shown in Figure 3c. As we argue below, the scheme shown in the left panel appears more compatible with steric considerations upon docking an RNA stem-loop in Ire1 RNase.

Our model suggests that the Ire1 RNase active site is unique and does not superimpose with other structurally characterized RNases. However, the arrangement of the catalytic residues in Ire1 and their interactions with the scissile phosphate resemble those in the active sites of ribonucleases T1 and A. By extension, we predict that the same mechanism applies to mammalian Ire1 and RNase L, the Ire1 homologue involved in the interferon response pathway, in which all relevant residues discussed here for Ire1 are strictly conserved.

### A possible mechanism of stem-loop RNA recognition by Ire1 RNase

The crystal structure of tRNA^Phe ^(PDB ID 1ehz) shows the stem-loop in a conformation that would preclude cleavage by Ire1 (Figure 5c, yellow trace). The RNA cleavage reaction imposes strict stereochemical requirements such that an incoming nucleophile can attack the phosphate in line with the leaving group. A similar stereochemical problem was encountered in studies of sarcin/ricin GAGA tetraloop cleavage by ribonucleases α-sarcin and restrictocin. A cocrystal structure of restrictocin with the sarcin/ricin stem-loop RNA suggested that scissile bases should undergo base flipping to position the incoming 2'OH in the 2'-endo conformation and to allow for in-line attack during catalysis [27]. Guided by this concept, we searched the PDB database for a tRNA^Phe ^structure, in which the loop guanosine at position 3 is flipped out such that its ribose ring adopts the 2'-endo conformation. We found that the crystal structure of tRNA^Phe ^complexed with the tRNA modifying enzyme MiaA [28] serves as a suitable model (Figure 5c, blue trace).

Accordingly, we used the stem-loop from the tRNA^Phe^·MiaA structure for rigid-body docking into Ire1 active site. As a restraint, we placed the scissile phosphate into the strongest electron density peak (Figure 5d) and the rest of the loop to allow sterically permissible interactions expected from the cleavage mechanism (I) in Figure 3c. The docked model has no backbone intersections or clashes of backbone atoms. By contrast, cleavage mechanism (II) could not be satisfied using this docking approach due to seemingly irresolvable interchain clashes. The resulting model predicts that only one stem-loop can bind to the Ire1 RNase dimer and that one RNase domain in the dimer cleaves RNA, whereas the other RNase monomer completes the RNA-contacting surface *in trans*, suggesting that both RNase monomers and both HLEs participate in recognition of the RNA stem-loop structure (Figure 5e).

Our model that the HLE of one RNase monomer contributes to RNA cleavage by the catalytic center of a different RNase monomer is functionally supported by the *in trans *complementation assay described above (Figure 4). This experiment demonstrates that cleavage by the active site of an RNase monomer is impaired upon removal of an RNA binding residue from an adjacent, non-catalytic monomer. Therefore, although Ire1 RNase has individual per-monomer catalytic centers, two RNase monomers are involved in RNA cleavage and apparently form a composite RNA binding surface created via dimerization. The high-order oligomerization of Ire1 completes the active site by stabilizing the highly dynamic HLE [5] in a conformation suitable for recognition of cognate RNA stem-loops. This mechanism could explain how self-assembly of Ire1 into an oligomer activates the RNase function.

### Ire1 cleavage of the tRNA^Phe ^anticodon loop

We found that, strikingly, Ire1 can recognize and cleave the anticodon stem/loop structure of tRNA^Phe ^in addition to the cognate splice site stem-loops of *HAC1 *and *XBP1 *mRNA. This observation redefines the known substrate specificity of Ire1 and suggests that additional stem-loops besides consensus sites in *HAC1 *and *XBP1 *mRNA could be cleaved by Ire1 RNase. Whether tRNA^Phe ^is an *in vivo *substrate of Ire1 remains to be investigated. In principle, tRNA destruction could serve to reduce the cell's translational capacity and thereby reduce protein influx into the ER, akin to protein kinase-like ER kinase (PERK) activation in metazoan cells. However, the scissile guanine in the anticodon of tRNA^Phe ^carries a post-transcriptional 2'-methoxy modification [29], which precludes cleavage of the modified tRNA^Phe ^pool by any RNase producing 2',3'-cyclic phosphate, such as Ire1, leaving only the unmodified pool as a possible substrate. It is tempting to speculate that the tRNA modification evolved to protect tRNA^Phe ^from cleavage by Ire1 and that regulation of the methylated pool of tRNA^Phe ^could provide a control point by which Ire1 may affect the translational capacity of cells.

## Conclusions

We investigated the mechanism of RNA cleavage by Ire1 RNase by combining computational, structural and quantitative biochemical approaches. This work experimentally determined the location of the catalytic center in Ire1 RNase and converged on a model of docking and cleavage of cognate RNA stem-loops. Histidine H1061 was defined as a key catalytic residue that is required in single copy in the twofold symmetric active site of the Ire1 RNase. Presented here *in trans *mutagenesis studies using (R1039A) and (R1039A, H1061N) Ire1 mutants, as well as electron density-guided *in silico *docking of an RNA hairpin with the scissile bond correctly aligned for catalysis suggest that the HLEs of two Ire1 monomers participate in stem-loop binding. This model intuitively predicts that only one RNA stem-loop binds to the dimer of Ire1 at a time (Figure 5e) whereas the high-order oligomerization of Ire1 enhances RNA recognition via allosteric stabilization of the HLE [5]. These findings identify the link between oligomerization of Ire1 and recognition of cognate RNA stem-loops, providing a glimpse of the central event in Ire1 signaling.

## Methods

### Experimental errors

Individual rate constants were determined from single-exponential fitting of time courses of RNA cleavage. Kinetic parameters were reproduced two or more times and were consistent between different days. Rate variations were typically within twofold; this uncertainty is small compared to the effects we describe as significant. Experimental errors are provided in the text and figure legends, when appropriate.

### Ire1 expression and purification

Ire1KR32 and its mutants were expressed as GST fusion proteins using pGEX-6P-2 plasmid (GE Healthcare, Waukesha, WI) and codon-compensated *Escherichia coli *(BL21-CodonPlus RIPL) (Stratagene, Santa Clara, CA). Expression was performed at room temperature for 4 h after IPTG induction. Cells were lysed using an Emulsiflex C-3 (Avestin Inc. Ottawa, Ontario, Canada) and proteins purified by affinity purification with subsequent cleavage of the GST tag by Prescission protease (GE Healthcare, Waukesha, WI). All protein mutants were fractionated by gel filtration on an S200 column to approximately 99% purity. Proteins stocks (10-20 mg/ml) were stored at -80°C in the presence of 5% glycerol.

### RNA substrates

RNA oligonucleotides were purchased from Dharmacon (Lafayette, CO), labeled at the 5'-terminus using T4 polynucleotide kinase and ^32^P-ATP (PerkinElmer, Waltham MA) and purified by 20% polyacrylamide gel electrophoresis (PAGE) that allowed a single-nucleotide resolution, as described previously [5].

### Ire1 RNase cleavage assay

Kinetics of RNA cleavage was conducted as described previously [5]. Typically, reactions were carried out in 10 μl volume at 30°C. Reactions were started by adding 1 μl of ^32^P-labeled RNA to 9 μl of premixture containing 20 mM 4-(2-hydroxyethyl)-1-piperazine-ethanesulfonic acid (HEPES) pH 7.4, 70 mM NaCl, 2 mM MgCl_2_, 4 mM dithiothreitol (DTT) and 5% glycerol. The reactions contained ≤ 1 pM radioactively (^32^P) labeled RNA and were conducted under single-turnover conditions (except for measurements of k_cat _and K_m _on Additional file 1, Figure S1, which were conducted under multiple-turnover conditions). Unless noted otherwise, Ire1 concentration was 3 μM. The enzyme concentration was determined from Ire1KR32 sequence using absorbance at 280 nm (e_280 _= 40.8 × 10^3 ^M/cm was calculated with BiochemLabSolutions ELN software, Princeton, NJ). Reactions were quenched at time intervals with 6 μl stop solution containing 10 M urea, 0.1% SDS, 0.1 mM ethylenediaminetetra-acetic acid (EDTA), 0.05% xylene cyanol and 0.05% bromophenol blue. Samples were analyzed by 10% to 20% PAGE, gels were scanned using Typhoon (Molecular Dynamics-GE Healthcare, Waukesha, WI) and quantified using ImageQuant (Molecular Dynamics-GE Healthcare, Waukesha, WI) and GelQuant.NET (BiochemLabSolutions, Princeton, NJ) programs. The data were plotted and fit in SigmaPlot to exponential curves to determine observed rate constants and to hyperbolic curves to determine binding constants.

### Ire1 oligomerization assay

Ire1 was in the reaction buffer containing 20 mM HEPES (pH 7.4), 70 mM NaCl, 2 mM MgCl_2_, 2 mM ADP, 4 mM DTT and 5% glycerol. Transparency of the samples containing higher concentrations of Ire1 visibly changed immediately upon adding the enzyme. Samples were allowed to sit for 15 min to allow for a complete Ire1 oligomerization. The optical density of the samples was subsequently measured at room temperature (22°C), at 500 nm on a UV-visible spectrophotometer Ultrospec 3300 Pro (Amersham-GE Healthcare, Waukesha, WI). OD_500 _was obtained after subtraction of baseline absorbance from the buffer free of Ire1. Sample absorbance did not change upon repeated scans of the same sample, indicating that the oligomerization reaches equilibrium under the conditions of the experiments.

### Ire1·(dCdCdGdCdAdG) complex crystallization

Ire1·(dCdCdGdCdAdG) complex was prepared by mixing Ire1KR32 and dCdCdGdCdAdG (IDT). When performed at relatively low NaCl concentration (300 mM or less) addition of the oligonucleotide causes profound precipitation of the oligomeric complex, as observed upon addition of ADP·Mg [5]. The oligonucleotide thus apparently promoted formation of Ire1KR32 oligomers analogous to ADP·Mg and to APY29. The precipitate readily redissolved upon slight increase in NaCl concentration, indicating that the oligomerization reaction was salt dependent and readily reversible. Crystallization was conducted in hanging drops using stock solution of premade complex containing Ire1KR32 (12 mg/ml) and 0.6 mM 2'-deoxy-CCGCAG. The well solution contained 0.12 M sodium citrate (pH 6.5), 7% polyethylene glycol (PEG) 3350 and 4% glucose. Single crystals grew overnight and were cryoprotected in well solution containing 25% ethylene glycol. The crystals belong to orthorhombic space group C222 distinct from the orthorhombic space group P2_1_2_1_2 previously reported for Ire1KR32 oligomer with bound APY29. Crystallization of a variety of other nucleotides and protein constructs was also tested but produced either inferior crystals or no crystals.

Diffraction from Ire1·(2'-deoxy-CCGCAG) crystals was collected on Beam Line BL 8.3.1 at the Advanced Light Source (Lawrence Berkeley National Laboratory, Berkeley, CA), at an X-ray wavelength of 1.115872 Å and an oscillation angle of 1°. The data were indexed, integrated and scaled using the XDS package [30] (Additional file 1, Table S1, Figure S5). A total of 5% of the reflections were marked as a test set (R_free_). A molecular replacement solution was found using PHASER [31] starting from monomer C of PDB ID 3fbv as a search model. Seven copies of monomer C were found in the asymmetric unit and the resulting 7-mer of Ire1KR32 was used for rigid body refinement in Phenix [32]. Simulated annealing was attempted and, expectedly, resulted in deterioration of statistics and excessive separation between R_work _and R_free _(Additional file 1, Table S1). Simulated annealing was therefore used only for calculation of omit maps (2000°C). Fourier σ_A_-weighted [33] F_obs_-F_calc _difference maps were used for interpretation of the parts of the model missing from the starting structure. Electron density and structure were analyzed and graphed in Coot [34] and PyMol (Schrödinger, San-Diego, CA). The sevenfold non-crystallographic symmetry (NCS) of the model was used to considerably increase quality of the electron density maps by averaging. However, all elements of Ire1 secondary structure were clearly visible without NCS. Coordinates have been deposited with the Protein Data Bank http://www.rcsb.org/ under accession number 3SDM.

### Crystallization of Ire1KR32(H1061N) oligomer

The H1061N mutant was crystallized and analyzed as described for the 3.2-Å structure of Ire1 [5]. Diffraction data were collected on the beamline 8.3.1 at the Advanced Light Source (ALS, Berkeley, CA) using wavelength 1.115879 Å. A total of 5% of reflections were marked as test set. The 3.2-Å structure [5] was used for molecular replacement. The H1061N mutation was introduced in Coot [34] prior to refinement. Rigid-body refinement and subsequent simulated annealing refinement (starting temperature 3200 K) using 14-fold non-crystallographic symmetry were performed in Phenix [32] (Additional file 1, Table S3). The resulting crystal structure had a good stereochemistry and no residues in disallowed regions on the Ramachandran plot. In order to reduce model bias, we also calculated an omit difference map from a model refined at 3200 K without the use of NCS restraints, from which a whole α-helix containing the H1061N residue was omitted prior to refinement. The resulting electron density clearly shows that the conformation of the H1061N mutant is identical to that of wild-type Ire1. Coordinates have been deposited with the Protein Data Bank http://www.rcsb.org/ under accession number 3SDJ.

## Authors' contributions

AVK designed and conducted the biochemical experiments and prepared protein and RNA constructs. AVK and PFE carried out Ire1 crystallization and diffraction data collection. JFM determined space groups and conducted initial diffraction data analyses. AVK and AAK carried out model refinement and building. CZ prepared synthetic small molecule modulators of Ire1. PW, RMS and KMS supervised the work. AVK and PW wrote the manuscript. All authors read and approved the final manuscript.

## Supplementary Material

Additional file 1 **Gepasi modelling files**. Compressed archive with Gepasi kinetics modeling files used in Figure S3. Click here for file

Additional file 2 **Supplementary information**. File with supplementary figures and tables. Click here for file

## References

[B1] Cox JS Walter P A novel mechanism for regulating activity of a transcription factor that controls the unfolded protein response Cell 1996 87 391 404 10.1016/S0092-8674(00)81360-4 8898193

[B2] Shamu CE Walter P Oligomerization and phosphorylation of the Ire1p kinase during intracellular signaling from the endoplasmic reticulum to the nucleus EMBO J 1996 15 3028 3039 8670804 PMC450244

[B3] Sidrauski C Cox JS Walter P tRNA ligase is required for regulated mRNA splicing in the unfolded protein response Cell 1996 87 405 413 10.1016/S0092-8674(00)81361-6 8898194

[B4] Mori K Ma W Gething MJ Sambrook J A transmembrane protein with a cdc2+/CDC28-related kinase activity is required for signaling from the ER to the nucleus Cell 1993 74 743 756 10.1016/0092-8674(93)90521-Q 8358794

[B5] Korennykh AV Egea PF Korostelev AA Finer-Moore J Zhang C Shokat KM Stroud RM Walter P The unfolded protein response signals through high-order assembly of Ire1 Nature 2009 457 687 693 10.1038/nature07661 19079236 PMC2846394

[B6] Lee KP Dey M Neculai D Cao C Dever TE Sicheri F Structure of the dual enzyme Ire1 reveals the basis for catalysis and regulation in nonconventional RNA splicing Cell 2008 132 89 100 10.1016/j.cell.2007.10.057 18191223 PMC2276645

[B7] Sidrauski C Walter P The transmembrane kinase Ire1p is a site-specific endonuclease that initiates mRNA splicing in the unfolded protein response Cell 1997 90 1031 1039 10.1016/S0092-8674(00)80369-4 9323131

[B8] Calvin K Xue S Ellis C Mitchell MH Li H Probing the catalytic triad of an archaeal RNA splicing endonuclease Biochemistry 2008 47 13659 13665 10.1021/bi801141q 19053288 PMC6296462

[B9] Yajima S Inoue S Ogawa T Nonaka T Ohsawa K Masaki H Structural basis for sequence-dependent recognition of colicin E5 tRNase by mimicking the mRNA-tRNA interaction Nucleic Acids Res 2006 34 6074 6082 10.1093/nar/gkl729 17099236 PMC1669751

[B10] Baker NA Sept D Joseph S Holst MJ McCammon JA Electrostatics of nanosystems: application to microtubules and the ribosome Proc Natl Acad Sci USA 2001 98 10037 10041 10.1073/pnas.181342398 11517324 PMC56910

[B11] Perez-Cano L Fernandez-Recio J Optimal protein-RNA area, OPRA: a propensity-based method to identify RNA-binding sites on proteins Proteins 78 25 35 10.1002/prot.22527 19714772

[B12] Korennykh A Pascal E Korostelev A Finer-Moore J Stroud R Walter P Cofactor-mediated conformational control in the bifunctional kinase/RNase Ire1 BMC Biol in press 10.1186/1741-7007-9-48 PMC3158555 21729334

[B13] Yusupova GZ Yusupov MM Cate JH Noller HF The path of messenger RNA through the ribosome Cell 2001 106 233 241 10.1016/S0092-8674(01)00435-4 11511350

[B14] Petry S Brodersen DE Murphy FVt Dunham CM Selmer M Tarry MJ Kelley AC Ramakrishnan V Crystal structures of the ribosome in complex with release factors RF1 and RF2 bound to a cognate stop codon Cell 2005 123 1255 1266 10.1016/j.cell.2005.09.039 16377566

[B15] Gonzalez TN Walter P Ire1p: a kinase and site-specific endoribonuclease Methods Mol Biol 2001 160 25 36 11265288 10.1385/1-59259-233-3:025

[B16] Quirk DJ Raines RT His ... Asp catalytic dyad of ribonuclease A: histidine pKa values in the wild-type, D121N, and D121A enzymes Biophys J 1999 76 1571 1579 10.1016/S0006-3495(99)77316-9 10049337 PMC1300133

[B17] Steyaert J Wyns L Functional interactions among the His40, Glu58 and His92 catalysts of ribonuclease T1 as studied by double and triple mutants J Mol Biol 1993 229 770 781 10.1006/jmbi.1993.1078 8433370

[B18] Dong B Niwa M Walter P Silverman RH Basis for regulated RNA cleavage by functional analysis of RNase L and Ire1p RNA 2001 7 361 373 10.1017/S1355838201002230 11333017 PMC1370093

[B19] Panov KI Kolbanovskaya EY Okorokov AL Panova TB Terwisscha van Scheltinga AC Karpeisky M Beintema JJ Ribonuclease A mutant His119 Asn: the role of histidine in catalysis FEBS Lett 1996 398 57 60 10.1016/S0014-5793(96)01173-8 8946953

[B20] Raines RT Ribonuclease A Chem Rev 1998 98 1045 1066 10.1021/cr960427h 11848924

[B21] Gonzalez TN Sidrauski C Dorfler S Walter P Mechanism of non-spliceosomal mRNA splicing in the unfolded protein response pathway EMBO J 1999 18 3119 3132 10.1093/emboj/18.11.3119 10357823 PMC1171393

[B22] Steyaert J Hallenga K Wyns L Stanssens P Histidine-40 of ribonuclease T1 acts as base catalyst when the true catalytic base, glutamic acid-58, is replaced by alanine Biochemistry 1990 29 9064 9072 10.1021/bi00490a025 1980211

[B23] Steyaert J Opsomer C Wyns L Stanssens P Quantitative analysis of the contribution of Glu46 and Asn98 to the guanosine specificity of ribonuclease T1 Biochemistry 1991 30 494 499 10.1021/bi00216a027 1899029

[B24] Steyaert J A decade of protein engineering on ribonuclease T1--atomic dissection of the enzyme-substrate interactions Eur J Biochem 1997 247 1 11 10.1111/j.1432-1033.1997.t01-1-00001.x 9249002

[B25] Loverix S Winquist A Stromberg R Steyaert J An engineered ribonuclease preferring phosphorothioate RNA Nat Struct Biol 1998 5 365 368 10.1038/nsb0598-365 9586998

[B26] Das SR Piccirilli JA General acid catalysis by the hepatitis delta virus ribozyme Nat Chem Biol 2005 1 45 52 10.1038/nchembio703 16407993

[B27] Yang X Gerczei T Glover L Correll CC Crystal structures of restrictocin-inhibitor complexes with implications for RNA recognition and base flipping Nat Struct Biol 2001 8 968 973 10.1038/nsb1101-968 11685244

[B28] Chimnaronk S Forouhar F Sakai J Yao M Tron CM Atta M Fontecave M Hunt JF Tanaka I Snapshots of dynamics in synthesizing N(6)-isopentenyladenosine at the tRNA anticodon Biochemistry 2009 48 5057 5065 10.1021/bi900337d 19435325 PMC2786004

[B29] Mucha P Szyk A Rekowski P Weiss PA Agris PF Anticodon domain methylated nucleosides of yeast tRNA(Phe) are significant recognition determinants in the binding of a phage display selected peptide Biochemistry 2001 40 14191 14199 10.1021/bi010978o 11714272

[B30] Kabsch W Automatic processing of rotation diffraction data from crystals of initially unknown symmetry and cell constants J Appl Cryst 1993 26 795 800 10.1107/S0021889893005588

[B31] McCoy AJ Phaser crystallographic software J Appl Cryst 2007 40 658 674 10.1107/S0021889807021206 PMC2483472 19461840

[B32] Adams PD Grosse-Kunstleve RW Hung LW Ioerger TR McCoy AJ Moriarty NW Read RJ Sacchettini JC Sauter NK Terwilliger TC PHENIX: building new software for automated crystallographic structure determination Acta Crystallogr D Biol Crystallogr 2002 58 1948 1954 10.1107/S0907444902016657 12393927

[B33] Read RJ Coefficients for maps using phases from partial structures with errors Acta Cryst 1986 A42 140 149

[B34] Emsley P Cowtan K Coot: model-building tools for molecular graphics Acta Crystallogr D Biol Crystallogr 2004 60 2126 2132 10.1107/S0907444904019158 15572765

