# Supplementary material for: Structural and functional basis for RNA cleavage by Ire1
Source: BMC Biol. 2011 Jul 6;9:47. doi: 10.1186/1741-7007-9-47 (PMC3149027; doi:10.1186/1741-7007-9-47)
Supplement: Additional file 2 — Supplementary information. File with supplementary figures and tables. [file 1741-7007-9-47-S2.DOC]

# Supplementary Figures


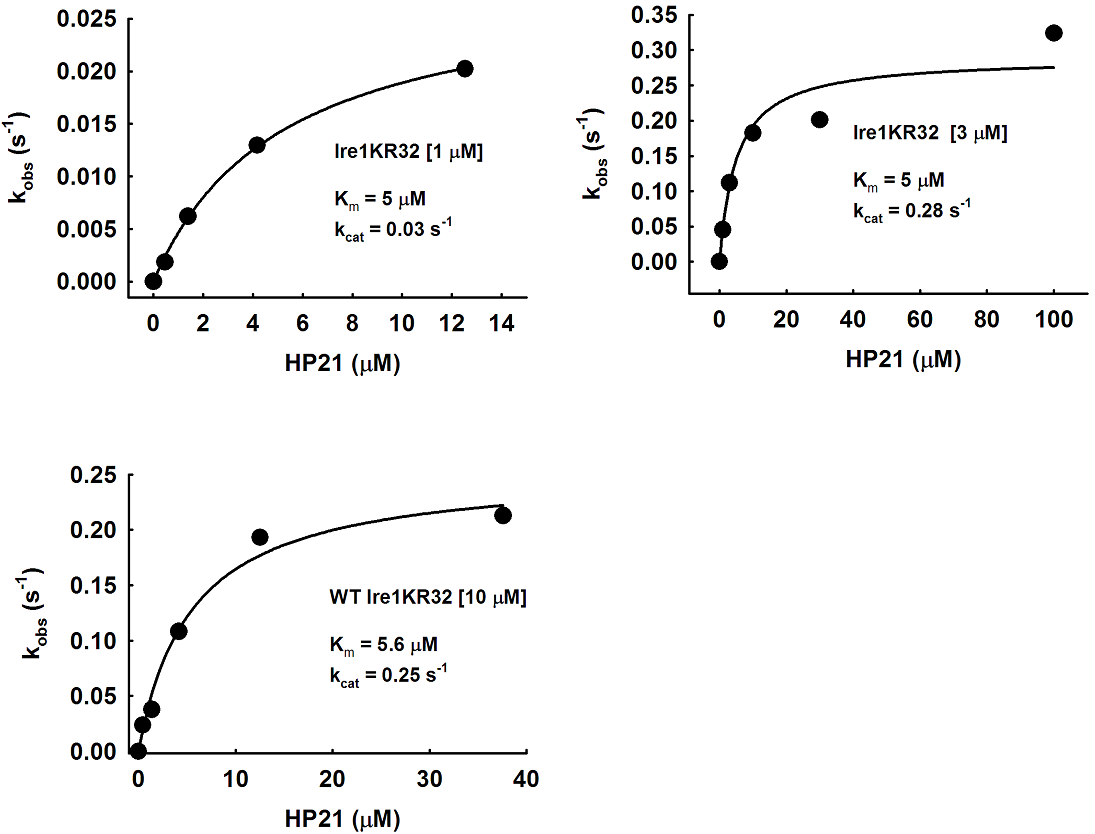


**Fig. S1** Measurements of k_cat_ and K_m_ for Ire1KR32 and HP21 at 1, 3 and 10 μM total Ire1KR32. Reactions were conducted as in Fig. 1c.

**
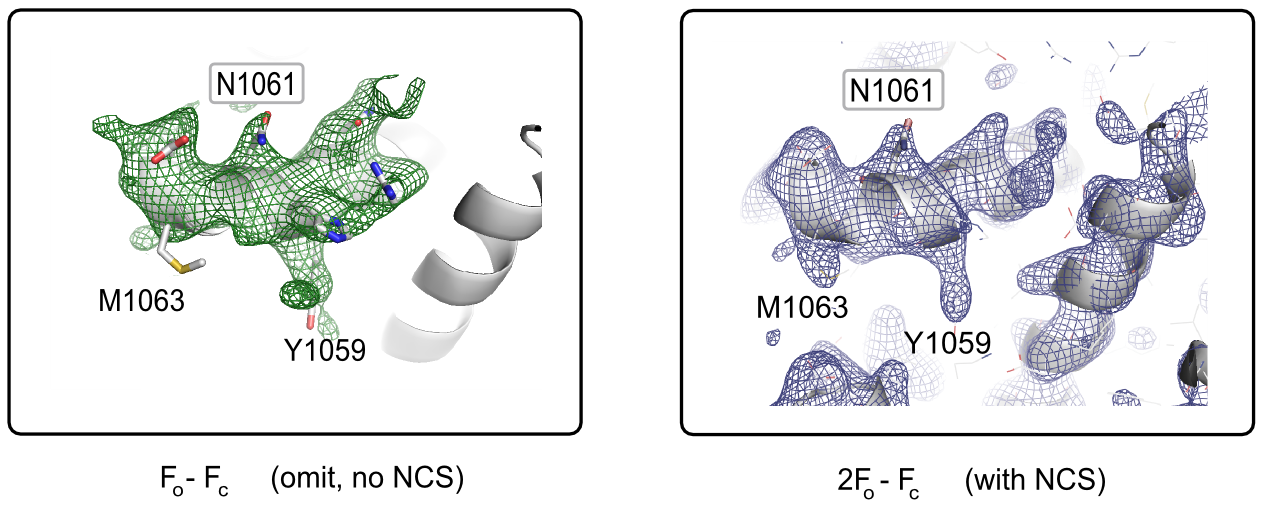
**

**Fig. S2** Simulated-annealing omit map F_o_-F_c_ for the H1061N mutant calculated without NCS (left). Contour level is 2σ. Simulated annealing (2000K) was conducted without NCS restraints and with residues 1056-1063 deleted from all 14 monomers in the asymmetric unit. 2F_o_-F_c_ map calculated with NCS from Fig. 2c is shown for comparison (right).


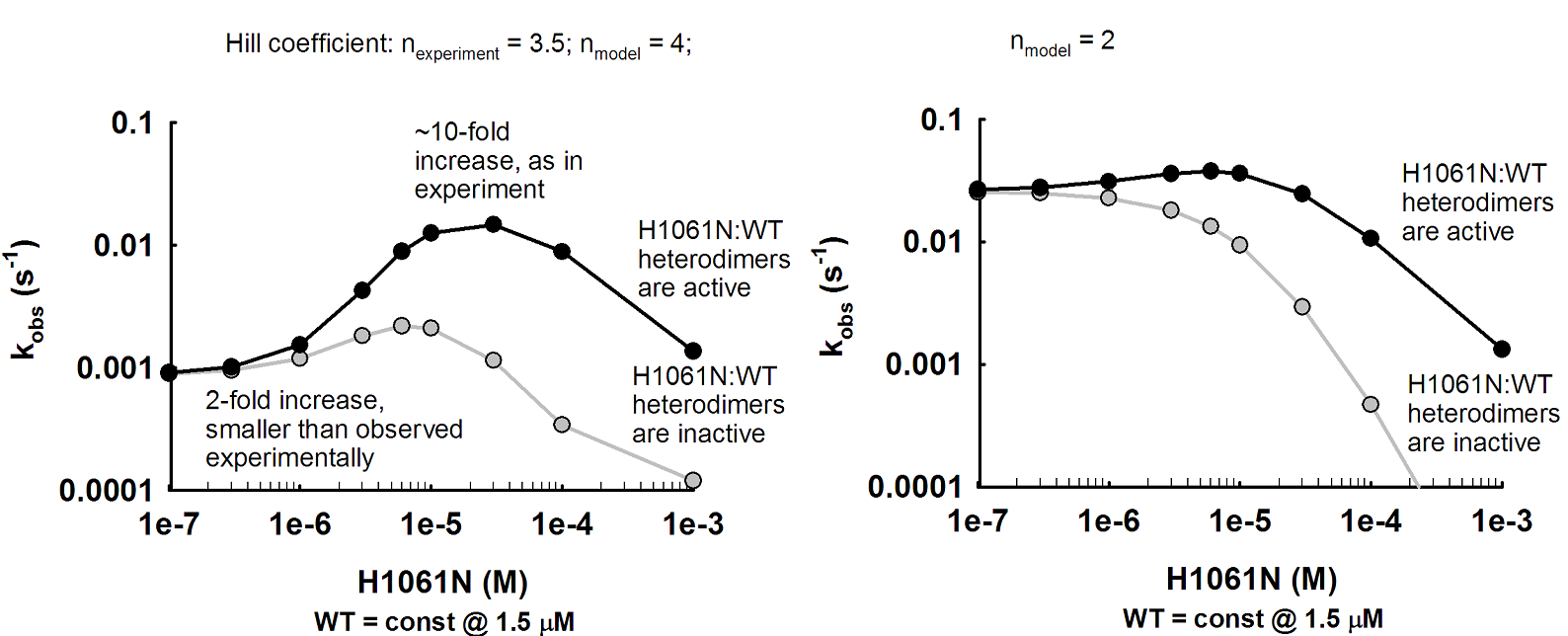

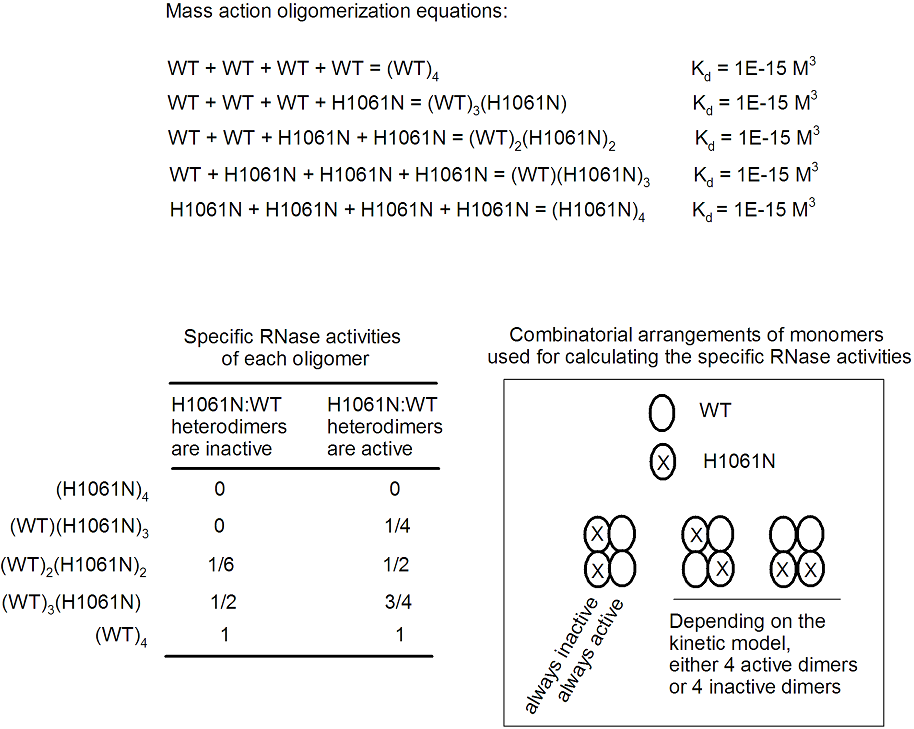


a

b

c

**Fig. S3** Modeling of in-trans activation of WT Ire1KR32 by Ire1KR32(H1061N) mutant using Gepasi 3.30 software. **a**, Mass action equations used in the model with n = 4. **b**, Specific activity of each of the tetramers for two scenarios: i) WT:H1061N dimer is active or ii) WT:H1061N dimer is inactive. **c**, Numeric differential equation modeling result. The ~10-fold activation phase is predicted only for RNase-active hetero-dimers, and for highly cooperative oligomerization with n = 4. Gepasi modeling files are included in supplementary information (Additional file 1).


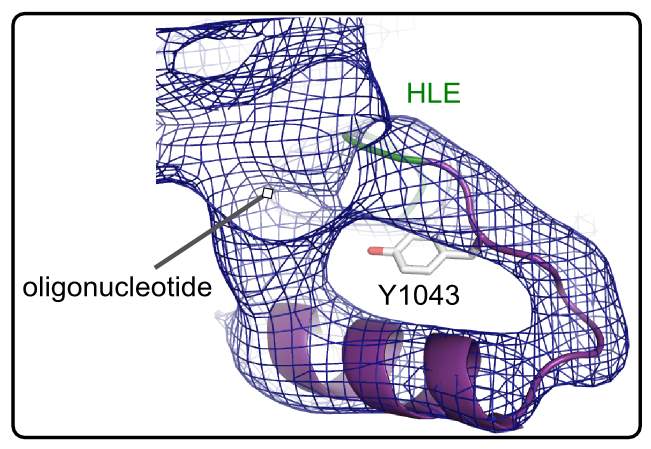


**Fig. S4** An alternative view of the simulated annealing omit map from Fig. 3b, calculated without NCS restraints. Contour level is 4σ.


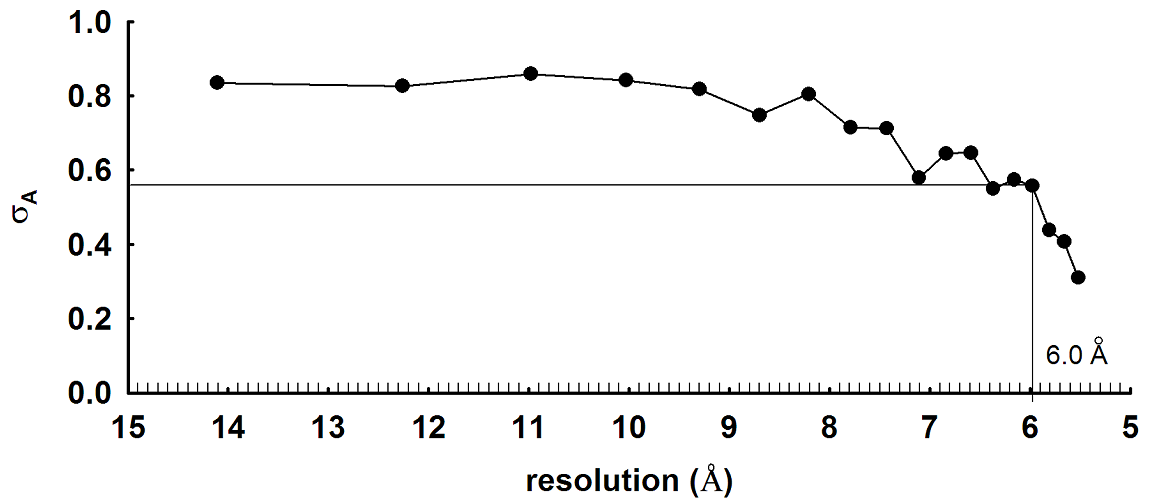


**Fig. S5** Graph of σ_A_ versus resolution for the C222 crystal [1-2]. Values remain significantly above zero at ≥ 6 Å.

**Supplementary Tables**

Table S1. Data collection and refinement statistics, C222 crystal with Ire1KR32 [3] and dCdCdGdCdAdG

| **Data Collection and Refinement** | Statistics | Subcategory | Value |
| --- | --- | --- | --- |
| Data collection | Cell dimensions | Space group | C222 |
|  |  | a,b,c (Å) | 91.67, 580.82, 177.99 |
|  |  | α, β, γ (^o^) | 90, 90, 90 |
|  |  | Resolution (Å) | 97-6.60 (6.8-6.60) |
|  |  | I/σ(I) | 16.62 (1.09) |
|  |  | R_pim_ ^&^ (%) | 4.4 (88.3) |
|  |  | n Reflections | 9402 (766) |
|  |  | Completeness (%) | 99.9 (99.9) |
| Refinement | **n atoms**  **(in 1 monomer)** | Protein | 3399 |
|  |  | Resolution (Å) | 97-6.60 |
|  | Rigid body(with NCS) | Angles (^o^) | 0.925 |
|  |  | Bonds (Å) | 0.006 |
|  |  | R_work_/R_free_ | 0.2863/0.3170 |
|  | **Simulated annealing (with NCS)** | t (^o^K) | 1000 |
|  |  | Angles (^o^) | 1.197 |
|  |  | Bonds (Å) | 0.007 |
|  |  | R_work_/R_free_ | 0.2731/0.3451 |

Refinement was done using rigid body protocol. Simulated annealing was not used in final analysis except for calculations of omit maps. Values for the highest resolution shell are given in parentheses.

^&^ Precision-indicating merging R-factor [4]

# Table S2. Protonatable neighboring residues of H1061

| **Residue** | **Distance from H1061 (Å) within the same monomer** | **Sequence conservation*** | **Effects of studied mutations^$^** |
| --- | --- | --- | --- |
| R1039 | 9.2 | K >> R > H, N, E, G, T, I, V | moderate;  ~1; ~10-fold^**^ |
| R1041 | 9.8 | R >> I, S, F, V, A | strong |
| **Y1043** | 8.0 | Y | strong;  ~10; ~100-fold^**^ |
| R1053 | 8.1 | R >> K | strong |
| **R1056** | 5.5 | R | strong |
| K1058 | 8.5 | K >> L > L, S, T | - |
| **H1061** | 0.0 | H | moderate;  ≥ 3**•**10^5^-fold^**^ |
| D1064 | 3.5 | D > E >> Q, S, N, T | - |

Bold shows residues invariant in all Ire1 and RNase L sequences

^$^combined qualitative data from [5-7]; "moderate": specific RNA cleavage products are seen on a gel in visibly lower amounts compared to WT enzyme; "strong": trace RNA cleavage or absence of cleavage band on a gel

* from alignment of 97 non-degenerate sequences of Ire1 + 13 sequences of

RNase L

** measured in this work; because the effects of the mutations depend on kinetic regime (saturating vs sub-saturating conditions), two values corresponding to ratios of k_2_ and k_2_/K_1/2_ are provided, when available

Table S3. Data collection and refinement statistics, P2_1_2_1_2 crystal of Ire1KR32Δ28•APY29 [3]

| **Data Collection and Refinement** | Statistics | Subcategory | Value |
| --- | --- | --- | --- |
| Data collection | Cell dimensions | Space group | P2_1_2_1_2 |
|  |  | a,b,c (Å) | 158.79, 163.450, 298.04 |
|  |  | α, β, γ (^o^) | 90, 90, 90 |
|  |  | Resolution (Å) | 49.65-3.65  (3.8-3.65)* |
|  |  | I/σ(I) | 8.87 (1.11) |
|  |  | R_pim_^&^ (%) | 2.3 (81.3) |
|  |  | n Reflections | 86608 (9758) |
|  |  | Completeness (%) | 99.7 (99.7) |
| Refinement |  | Resolution (Å) | 3.65 |
|  | **n atoms** | Protein | 47990 |
|  |  | Ligand (APY29) | 350 |
|  |  | Water | 0 |
|  |  |  |  |
|  | R.m.s deviations | Angles (^o^) | 1.027 |
|  |  | Bonds (Å) | 0.006 |
|  |  | R_work_/R_free_ | 0.2485/0.2873 |

* Values for the highest resolution shell are given in parentheses

^&^ Precision-indicating merging R-factor [4]

References

1. Brunger AT, DeLaBarre B, Davies JM, Weis WI: **X-ray structure determination at low resolution**. *Acta Crystallogr D Biol Crystallogr* 2009, **65**(Pt 2):128-133.

2. Ling H, Boodhoo A, Hazes B, Cummings MD, Armstrong GD, Brunton JL, Read RJ: **Structure of the shiga-like toxin I B-pentamer complexed with an analogue of its receptor Gb3**. *Biochemistry* 1998, **37**(7):1777-1788.

3. Korennykh AV, Egea PF, Korostelev AA, Finer-Moore J, Zhang C, Shokat KM, Stroud RM, Walter P: **The unfolded protein response signals through high-order assembly of Ire1**. *Nature* 2009, **457**(7230):687-693.

4. Weiss MS: **Global indicators of X-ray data quality**. *J Appl Cryst* 2001, **34**:130-135.

5. Dong B, Niwa M, Walter P, Silverman RH, Walter HJ, McMahon T, Dadgar J, Wang D, Messing RO, Walter MH *et al*: **Basis for regulated RNA cleavage by functional analysis of RNase L and Ire1p**. *RNA* 2001, **7**(3):361-373.

6. Tirasophon W, Lee K, Callaghan B, Welihinda A, Kaufman RJ: **The endoribonuclease activity of mammalian IRE1 autoregulates its mRNA and is required for the unfolded protein response**. *Genes Dev* 2000, **14**(21):2725-2736.

7. Lee KP, Dey M, Neculai D, Cao C, Dever TE, Sicheri F: **Structure of the dual enzyme Ire1 reveals the basis for catalysis and regulation in nonconventional RNA splicing**. *Cell* 2008, **132**(1):89-100.
